# Development of Prone Position Ventilation Device and Study on the Application Effect of Combined Life Support Technology in Critically Ill Patients

**DOI:** 10.1155/2024/5812829

**Published:** 2024-08-19

**Authors:** Yufeng Li, Qiaoqiao Hu, Wenjie Wang, Changhong Du, Siwen Fan, Linlin Xu, Songmei Li, Bei Chen

**Affiliations:** ^1^ Department of Intensive Care Unit Xuzhou Central Hospital, Xuzhou 221009, China; ^2^ Department of Operating Room Xuzhou Central Hospital, Xuzhou 221009, China

## Abstract

**Objective:**

This study aims to evaluate a novel prone position ventilation device designed to enhance patient safety, improve comfort, and reduce adverse events, facilitating prolonged tolerance in critically ill patients.

**Methods:**

A randomized controlled trial was conducted on 60 critically ill patients from January 2020 to June 2023. Of which, one self-discharged during treatment and another was terminated due to decreased oxygenation, leaving an effective sample of 58 patients. Patients were allocated to either a control group receiving traditional prone positioning aids (ordinary sponge pads and pillows) or an intervention group using a newly developed adjustable prone positioning device. A subset of patients in each group also received life support technologies such as extracorporeal membrane oxygenation (ECMO) and continuous renal replacement therapy (CRRT). We assessed prone position ventilation tolerance, oxygen saturation increments postintervention, duration of prone positioning, CRRT filter lifespan, and the incidence of adverse events.

**Results:**

The intervention group exhibited significantly longer average tolerance to prone positioning (16.6 hours vs. 8.3 hours, *P* < 0.001 with a difference of 8.3 (4.4, 12.2) hours), higher increases in oxygen saturation postventilation (9% vs. 6%, *P* < 0.001 with a difference of 3.0 (1.5, 4.5)), and reduced time required for medical staff to position patients (11.7 min vs. 21.8 min, *P* < 0.001 with a difference of −10.1 (−11.9, −8.3)). Adverse events, including catheter displacement or blockage, facial edema, pressure injuries, and vomiting or aspiration, were markedly lower in the intervention group, with statistical significance (*P* < 0.05). In patients receiving combined life support, the intervention group demonstrated improved catheter blood drainage and extended CRRT filter longevity.

**Conclusion:**

The newly developed adjustable prone ventilation device significantly improves tolerance to prone positioning, enhances oxygenation, and minimizes adverse events in critically ill patients, thereby also facilitating the effective application of life support technologies.

## 1. Introduction

Acute respiratory distress syndrome (ARDS) presents a formidable challenge in critical care, necessitating innovative approaches to mechanical ventilation [1]. Among these, prone position ventilation (PPV) has emerged as a pivotal intervention [2]. PPV entails repositioning the patient from supine to prone, a maneuver that redistributes pulmonary perfusion and optimizes ventilation-perfusion matching, thereby enhancing alveolar recruitment and improving oxygenation [3]. The efficacy of PPV in ARDS has been substantiated through extensive research, indicating a significant enhancement in patient oxygenation and a reduction in mortality risk ratios to 0.85 (95% confidence interval: 0.71–1.01) compared to supine ventilation [4].

The relevance of PPV extends beyond traditional ARDS management; it has shown promising results in the treatment of COVID-19-induced ARDS, contributing to the amelioration of patient outcomes [5]. Current therapeutic protocols advocate for extended durations of PPV, emphasizing its critical role in severe pulmonary conditions [6].

Despite its proven benefits, the implementation of PPV remains challenging, particularly in conjunction with life support modalities such as extracorporeal membrane oxygenation (ECMO) and continuous renal replacement therapy (CRRT). The intricacies of patient care in the prone position are amplified by the presence of various high-risk catheters and the demands of monitoring complex support systems [7–10]. The incidence of prone position-related complications, including pressure ulcers (reported in up to 40% of patients), reflux aspiration, and catheter-related issues, further complicates patient management and underscores the need for specialized equipment [1–3].

Several devices have been developed to facilitate safe prone ventilation management, including various types of cushions and pads designed to reduce pressure on vulnerable areas. For example, ordinary sponge pads and pillows are commonly used as positioning aids but often fail to adequately prevent pressure injuries and ensure patient comfort over prolonged periods [11]. Other more advanced devices include foam positioners and air-filled cushions, which aim to distribute pressure more evenly and reduce the risk of skin breakdown [12]. However, these devices have their limitations, including difficulties in adjusting to different patient anatomies and the requirement for frequent repositioning by medical staff [13].

A recent study by Douglas et al. [14] on the safety and outcomes of prolonged prone position ventilation for COVID-19-induced hypoxemic respiratory failure revealed significant adverse events. Among 61 patients treated with prone position ventilation, 71.7% developed ventral pressure wounds and 95.1% experienced limb weakness, with 8.2% suffering from brachial plexus palsies. These findings highlight the urgent need for more effective and safer prone positioning solutions.

In response to these challenges, this study introduces an adjustable prone positioning device tailored to the needs of critical care. Designed to mitigate complications and streamline patient management, the present new device was designed to provide better pressure distribution, ease of adjustment, and enhanced patient safety compared to existing solutions. The findings from its clinical application, including detailed statistics on adverse events and comparative effectiveness, are detailed in the subsequent sections.

## 2. Methods

This study was conducted as a randomized controlled trial (RCT) with a 1 : 1 allocation ratio. The trial aimed to evaluate the efficacy and safety of a novel adjustable prone position ventilation device compared to traditional prone positioning aids in critically ill patients. Patients were randomly assigned to either the intervention group, using the new adjustable prone positioning device, or the control group, utilizing standard prone positioning aids. The randomization process for this study was conducted using a computer-generated random sequence to ensure unbiased assignment of participants to either the intervention group or the control group. The allocation ratio was 1 : 1. The randomization was stratified to ensure a balanced distribution of key baseline characteristics between the groups. This study was reported in accordance with the Consolidated Standards of Reporting Trials (CONSORT) guidelines (as shown in Appendix I). The CONSORT checklist, which outlines the key elements required for transparent and complete reporting of randomized controlled trials, has been included as a supplementary document to this manuscript.

### 2.1. Ethical Considerations

This study was conducted following the ethical principles outlined in the Declaration of Helsinki. Ethical approval was obtained from the Institutional Review Board (IRB) of Xuzhou Central Hospital, with the approval number XZCH102454. All patients or their legal representatives provided written informed consent prior to participation in the study.

### 2.2. Device Fabrication

#### 2.2.1. Construction of the Adjustable Prone Position Ventilation Device

**Figure 1:**
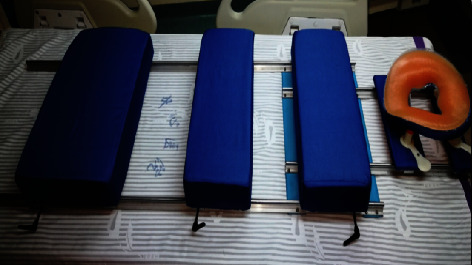
The novel adjustable prone position ventilation apparatus.

*Materials*. The device was assembled from high-precision components (Figure 1), including two 1400 mm-length dual-axis precision guide rails, two 600 mm-length dual-axis precision guide rails, sixteen multiwheel locking sliders for rail compatibility, three 7075-T6 aviation-grade aluminum plates (560 mm × 40 mm × 4 mm each), one 7075-T6 aviation-grade aluminum plate (300 mm × 30 mm × 4 mm), two support boards (560 mm × 300 mm × 10 mm and 560 mm × 200 mm × 10 mm) with latex pads, and one perforated support board (300 mm × 300 mm × 10 mm) with a face-shaped prone pillow, as shown in Figure 1.

*Fabrication Process*. The longer aluminum plates were used to join the 1400 mm guide rails. The 600 mm guide rails were attached perpendicularly to one end of the 1400 mm rails, forming the base of the device. Locking sliders were affixed to the support boards, which were then mounted onto the base rails. The latex pads were secured to the boards to create cushioned supports for various body regions. The device's functionality and safety were validated through simulation, innovation processes, and peer expert review.

#### 2.2.2. Device Utilization Protocol

**Figure 2:**
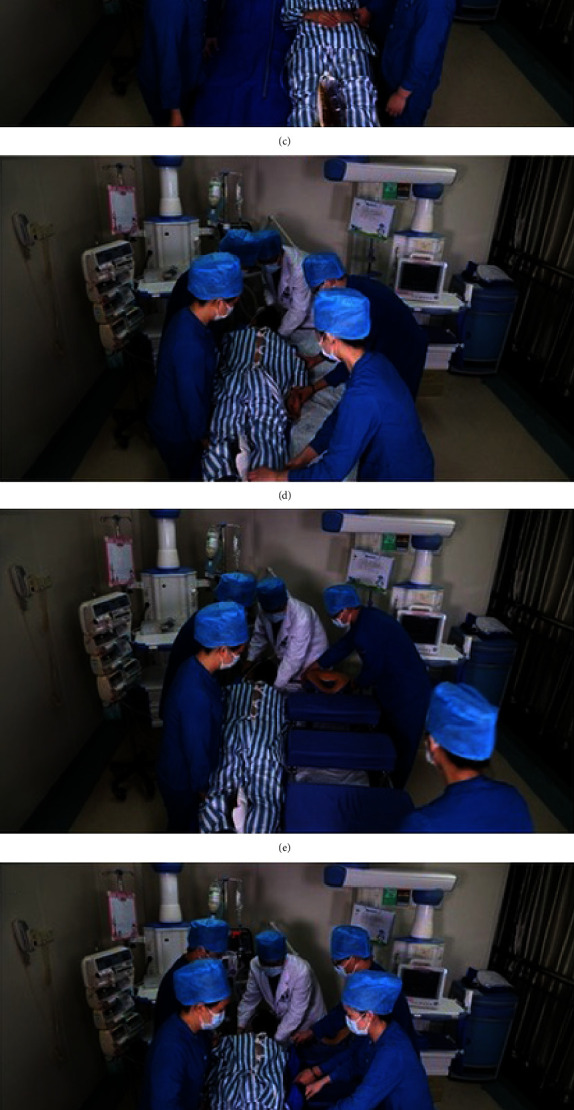
Sequential steps for prone position ventilation in patients. *Notes*. (a) Preparation: before repositioning, the patient's various catheters and lines are organized to prevent pressure or displacement. (b) Positioning: the patient is moved to the side of the bed to facilitate turning. (c) Bed preparation: a matching bed sheet is spread easily to the other side of the bed to assist in turning the patient. (d) Initial turning: the patient is turned over to the bed and temporarily placed in a prone position. (e) Ventilator placement: a prone ventilator is positioned beside the patient to ensure readiness for connection. (f) Final positioning: using the bed, the patient is moved into the prone position on the ventilation device. (g) Securing: the bottom buckle is opened, and the bed is removed to secure the patient on the prone ventilation device. (h) Connection and arrangement: ECG monitoring is connected, various lines are arranged, and prone position ventilation is completed.

**Figure 3:**
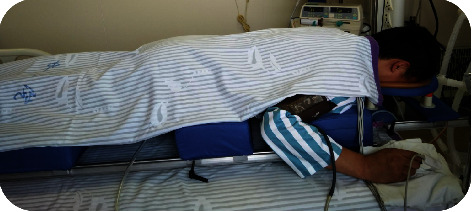
Prone position ventilation status.

**Figure 4:**
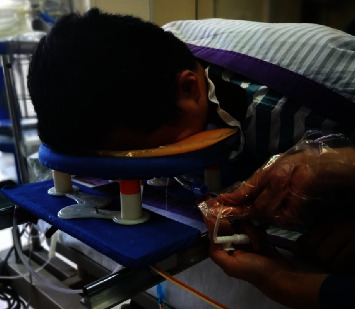
Application of the prone ventilation apparatus.

As is shown in Figures 2 and 3, the detailed operation steps are as follows: four medical staff members are positioned on either side of the patient's bed, with a doctor or respiratory therapist at the head of the bed, meticulously organizing and placing the endotracheal tube, ventilator circuit, intravenous lines, and drainage tubes. The patient is shifted to one side of the bed, and a prone positioning device is placed on the other side. The headrest, chest pad, iliac crest pad, and leg pads are adjusted to align with the patient's proportions and then the handle is turned clockwise to lock the sliders in place. Five medical personnel rotate the patient onto the device in a rolling motion so that the patient's head, shoulders, iliac crest, and legs are positioned on the respective pads, with the head, part of the chest, and abdomen suspended in the air. Both arms are naturally bent and placed on either side of the device, while the feet dangle naturally, thus facilitating prone ventilation (Figure 4). After prone positioning is concluded, the staff return to their initial positions, reorganize the tubing, lift the device with the patient to one side of the bed, flip the patient back to a supine position, remove the device, and then clean and disinfect the support pad covers.

### 2.3. Clinical Application

#### 2.3.1. Participant Selection

Sixty critically ill patients who received prone ventilation from January 2020 to June 2023 were selected. Inclusion criteria were as follows: critically ill patients requiring prone ventilation, including severe pneumonia, novel coronavirus infection, myocarditis, renal failure, viral pneumonia, drowning, and respiratory failure. Among the included cases, 20 patients received extracorporeal life support techniques such as ECMO and CRRT. Exclusion criteria were as follows: hemodynamic instability; increased intracranial pressure; acute phase of hemorrhagic disease; spinal or cervical injury; restrictive positioning; thoracoabdominal surgery or severe trauma; pregnancy; facial surgery or trauma; intolerance to prone positioning; rapid desaturation after prone positioning; severe arrhythmias; and patients with recently placed cardiac pacemakers. Of the 60 patients, one patient opted to leave the hospital against medical advice while not actively undergoing mechanical ventilation. Another patient was withdrawn from the study following a significant decrease in oxygenation levels after prone positioning, which was predetermined as an exclusion criterion for continued participation. Consequently, the study proceeded with an effective sample of 58 patients. These were equally divided into two groups, with 29 patients in the traditional group and 29 in the innovative group.

#### 2.3.2. Sample Size Calculation

We aimed to detect a clinically significant difference in the primary outcome, which was the average tolerance to prone positioning. Based on previous studies and clinical expertise, we estimated a difference of 8 hours in prone positioning tolerance between the intervention and control groups, with a standard deviation of 6 hours. Using a two-sided significance level of 0.05 and a power of 80%, the sample size calculation was performed using the following formula for comparing two independent means:

*n*=((*Z*_*α*/2_+*Z*_*β*_)/(Δ/*σ*))^2^, *Z*_*α*/2_ is the critical value for a two-sided test at the 0.05 significance level (1.96). *Z*_*β*_ is the critical value for a power of 80%. Δ is the estimated difference in means (8 hours) and *σ* is the standard deviation (6 hours). Plugging in these values, *n* ≈ 20. To account for potential dropouts and to ensure the robustness of our results, we rounded up and aimed to recruit at least 25 patients per group, resulting in a total sample size of 50 patients. This approach ensured that even with a small number of dropouts, the study would remain adequately powered to detect the expected differences.

#### 2.3.3. Intervention Strategy

The traditional group was managed with standard care, while the innovative group utilized the new device. This allowed a direct comparison of outcomes attributable to the device usage.

*(1) Standard of Care*. Patients in the control group received traditional prone positioning aids, which included the use of ordinary sponge pads and pillows. These aids are commonly used in clinical settings to facilitate prone positioning in critically ill patients. The standard of care for prone positioning involves (1) positioning aids: traditional sponge pads and pillows were used to support the patient in the prone position, ensuring adequate comfort and stability. (2) Monitoring and adjustment: continuous monitoring of patient comfort and respiratory parameters was performed, with adjustments made as necessary to maintain optimal positioning. (3) Safety measures: standard safety protocols were followed to minimize the risk of pressure ulcers, reflux aspiration, and catheter-related issues, which are common complications associated with prone positioning. This group is further referred to as the “traditional group” in subsequent sections and tables.

*(2) Intervention Group*. The intervention group used a newly developed adjustable prone positioning device designed to enhance patient safety, comfort, and tolerance to prolonged prone positioning. This innovative device includes features such as adjustable supports and enhanced padding to reduce the risk of complications associated with traditional prone positioning aids. The intervention for this group involved (1) adjustable positioning device: the new device provided customizable support and padding to optimize patient comfort and stability during prone positioning. (2) Enhanced monitoring: in addition to standard monitoring, specialized sensors integrated into the device provided real-time feedback on patient positioning and pressure distribution. (3) Safety protocols: enhanced safety protocols were implemented to further reduce the incidence of adverse events, including pressure ulcers, reflux aspiration, and catheter-related issues. This group is further referred to as the “innovative group” in subsequent sections and tables.

#### 2.3.4. Data Collection Procedures

Data collection was conducted by a team of trained research nurses and clinical staff. Each member of the data collection team received specific training on the study protocol and data entry procedures to ensure consistency and accuracy. The data collection forms utilized in this study are shown in Appendix II.

1. *Patient Enrollment and Baseline Data*: upon enrollment, baseline demographic and clinical data were collected. This included patient age, gender, BMI, heart rate (HR), mean arterial pressure (MAP), APACHE II score, SOFA score, and acute lung injury score.
2. *Procedure Monitoring*: during the study, the research nurses and clinical staff monitored the implementation of PPV, including duration and frequency, as well as any complications arising from the procedure. Ventilator parameters such as tidal volume, respiratory rate, and positive end-expiratory pressure (PEEP) were recorded regularly.
3. *Complication Assessment*: complications were assessed using predefined criteria and guidelines. Pressure injuries were evaluated following the European Pressure Ulcer Advisory Panel (EPUAP) guidelines, and the occurrence of catheter migration, occlusion, hemodynamic disturbances, vomiting, aspiration, and head and face edema were meticulously documented.
4. *Data Entry and Verification*: All collected data were entered into a secure electronic database. Data entry was verified by a second member of the team to minimize errors. Regular audits were conducted to ensure data integrity and compliance with the study protocol.

#### 2.3.5. Outcome Measures

*(1) The Average Tolerance Time of PPV Patients in Two Groups*. To evaluate the average tolerance duration of prone position ventilation (PPV) in patients from two groups, we recorded the period from the initiation of prone positioning to the reversion to the supine position, triggered either by reaching the target time or due to adverse events.

*(2) The Time Required for Paramedics to Position the Patient in a Prone*. This metric involves timing the paramedics as they shift the patient from a supine to a prone position, starting from the preparation of ventilation equipment.

*(3) Improved Oxygen Saturation Post-Prone Positioning*. We monitored the trends in oxygen saturation at 1 hour, 3 hours, 6 hours, and at the end of the prone positioning period in both groups.

*(4) Incidence of Poor Blood Flow in the Circuit and Lifespan of the Continuous Renal Replacement Therapy (CRRT) Filter during PPV Combined with Extracorporeal Life Support Techniques*. Parameters such as ECMO, circuit vibration at the CRRT blood drainage end, and alarms triggered by exceeding arterial pressure limits were used as indicators of poor blood drainage. The lifespan of the CRRT filter was defined as the duration from the start of its use to the point when transmembrane pressure exceeded the limit, necessitating the filter's replacement.

*(5) PPV Complication Rates in Both Groups*. Complications of PPV encompassed issues like catheter migration or occlusion, hemodynamic disturbances, pressure injuries (including redness, blistering, ulceration, and necrosis), vomiting or aspiration, and edema of the head and face. The complication rate for PPV was calculated based on the number of complications per valid sample size in the observation or control group.

### 2.4. Statistical Analysis

Data were collected in Excel and analyzed using SPSS 26.0. Quantitative data were described with means ± standard deviation or median (interquartile range) where appropriate. Categorical data were expressed as frequency (%). The *t*-test or Mann–Whitney *U* test was used for mean comparisons and the Chi-square or Fisher's exact test for categorical data. Significance was set at *P* < 0.05 and high significance at *P* < 0.01. For all primary and secondary outcome measures, we also calculated 95% confidence intervals (CIs) to assess the precision of the estimated effects.

## 3. Results

### 3.1. Basic Characteristics

In this comparative study, demographic and clinical characteristics between the traditional group (*N* = 29) and the innovative group (*N* = 29) were analyzed (Table 1). The gender distribution was similar between groups, with 75.86% males in the traditional group and 72.41% in the innovative group (*t* = 2.463 and *P*=0.373). Age was comparable between groups (traditional: 63.5 ± 9.7 years; innovative: 63.8 ± 9.1 years; *t* = 3.646 and *P*=0.271). Body Mass Index (BMI) also showed no significant difference (traditional: 25.66 ± 5.1 kg/m^2^; innovative: 26.17 ± 4.6 kg/m^2^; *t* = 4.172 and *P*=0.246). Heart rate (HR) and mean arterial pressure (MAP) were statistically similar (HR: *t* = 2.673 and *P*=0.527; MAP: *t* = 0.836 and *P*=0.863). APACHE II scores, indicating the severity of disease, were not significantly different (traditional: 19.21 ± 1.76; innovative: 19.26 ± 2.15; *t* = 0.794 and *P*=0.917), and the Sequential Organ Failure Assessment (SOFA) scores were closely matched (traditional: 7.52 ± 2.64; innovative: 7.69 ± 2.37; *t* = 0.483 and *P*=0.956). Acute lung injury scores were comparable (traditional: 7.61 ± 3.91; innovative: 8.15 ± 3.65; *t* = 1.463 and *P*=0.748) as were lactate levels (traditional: 6.31 ± 2.65 mmol/L; innovative: 5.91 ± 3.26 mmol/L; *t* = 4.726 and *P*=0.217). Ventilator settings, such as tidal volume and respiratory rate, showed no significant differences (tidal volume: *t* = 3.747 and *P*=0.266; respiratory rate: *t* = 6.262 and *P*=0.173), and positive end-expiratory pressure (PEEP) was also similar between groups (traditional: 10.38 ± 4.13 cm H_2_O; innovative: 11.05 ± 5.39 cm H_2_O; *t* = 3.733 and *P*=0.274). These results suggest no statistically significant differences in the measured parameters between the traditional and innovative groups, indicating that the basic characteristics were comparable in the two groups.

### 3.2. Comparative Analysis of the Average Tolerance Duration for PPV, Average Increase in SaO_2_ Post-PPV, and Average Duration of Medical Personnel's Operation between the Two Groups

Patients in the innovative group exhibited an average tolerance duration of approximately 10 hours longer than those in the traditional group, meeting the daily minimum of 16 hours of prone positioning as recommended by various guidelines and consensus statements. The average increase in SaO_2_ post-PPV in the innovative group was 3% higher than that in the traditional group, indicating a more significant improvement in patients' oxygenation, *P* < 0.001, which is statistically significant. The average duration of medical personnel's operation for implementing PPV in the innovative group was reduced by 10 minutes compared to the traditional group, *P* < 0.001, which is also statistically significant (Table 2).

**Figure 5:**
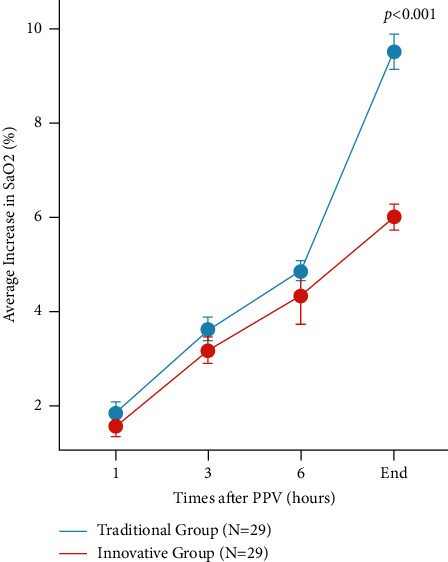
Average increase in SaO_2_ following PPV.

In our randomized controlled trial, we evaluated the efficacy of two PPV protocols—termed “traditional” and “innovative”—in enhancing arterial oxygen saturation (SaO_2_) over time. As illustrated in Figure 5, both groups commenced with comparable SaO_2_ levels. However, over successive time intervals (1, 3, and 6 hours), we observed a progressive increase in SaO_2_ in both cohorts. Notably, by the end of the monitoring period, the innovative group exhibited a significantly higher average increase in SaO_2_ compared to the traditional group (*P* < 0.001), suggesting that the innovative PPV approach may be more effective in improving oxygenation in this patient population. These findings have important implications for clinical practices in respiratory care, indicating that the innovative PPV protocol could potentially be a superior strategy for managing patients requiring ventilatory support (Figure 5).

#### 3.2.1. Comparison of the Incidence of Complications during PPV between the Two Groups

The incidence of complications such as catheter displacement or obstruction, facial edema, pressure-related injuries, and vomiting or aspiration was significantly lower in the innovative group compared to the traditional group, with *P* < 0.05, indicating statistical significance. The rate of hemodynamic disturbances was comparable between the groups, with no statistical significance (Table 3).

#### 3.2.2. Comparison of Catheter Efficiency and CRRT Filter Lifespan in Patients Undergoing PPV with ECMO and CRRT

The frequency of suboptimal catheter blood withdrawal in the innovative group was significantly lower compared to the traditional group during ECMO and CRRT procedures, with *P* < 0.001, denoting statistical significance. Furthermore, the average lifespan of CRRT filters in the innovative group exceeded that of the traditional group, with *P* < 0.001, which is statistically significant (Table 4).

## 4. Discussion

The study found several key findings with significant implications for the management of critically ill patients requiring prone ventilation. Notably, the introduction of a novel adjustable prone position ventilation device resulted in markedly improved patient tolerance to prone positioning, as evidenced by longer duration of prone positioning without discomfort. In conjunction with this, patients experienced enhanced oxygen saturation levels, indicative of improved pulmonary function. The device also benefited medical staff, as it reduced the time required for patient positioning, thereby optimizing workflow efficiency. In addition, when used in patients receiving CRRT, the device was associated with an extended lifespan of CRRT filters, suggesting potential cost-effectiveness and clinical utility in a critical care setting. These findings collectively highlight the multifaceted benefits of the innovative device, which may revolutionize current practices in the treatment of ARDS and similar respiratory conditions.

Research by Guérin C and colleagues [11] has revealed that prone ventilation exceeding 16 hours daily is imperative for the effective amelioration of patients' oxygenation and the correction of hypoxemia. Utilizing conventional methods involving standard foam pads and pillows for prone positioning, patients exhibited an average tolerance duration of 8.3 hours, with prolonged immobility in the same posture leading to fatigue or even restlessness. The innovative prone ventilation apparatus, with its sliding rail-compatible support pads, allows for adjustments to accommodate the patient's unique body structure, catering to various body proportions. In addition, it enables periodic repositioning of the support pads to alter the pressure points on the patient, thereby enhancing comfort. Patients utilizing this cutting-edge prone ventilation device achieved an average tolerance duration of 16.6 hours.

When employing conventional prone positioning techniques with ordinary foam pads and pillows, the patient's chest and abdomen often serve as the primary support points, which cannot be suspended, leading to compression and subsequent respiratory compromise [12]. In contrast, the advanced prone ventilation device, by partially suspending the anterior chest and shoulder regions as well as the iliac crest, facilitates mechanical ventilation with a significant portion of the chest and abdomen in a suspended state. This reduces the transpulmonary pressure and driving pressure, thereby increasing pulmonary compliance and consequently improving oxygenation more effectively. In addition, the innovative design allows for the head to be suspended, aiding in the drainage and aspiration of secretions and reducing peak airway pressure. The increase in SaO2 following PPV was on average 9% for the innovative group compared to 6% for the conventional group, with *P* < 0.05, indicating statistical significance.

A randomized trial involving 342 patients revealed that patients receiving prone ventilation were more susceptible to the following adverse events compared to traditional supine ventilation: increased sedation or muscle relaxant requirements (80% vs. 56%), hypotension or arrhythmias (72% vs. 55%), transient desaturation of oxygenated hemoglobin (64% vs. 51%), airway obstruction (51% vs. 34%), vomiting (29% vs. 13%), loss of venous access (16% vs. 4%), and displacement of endotracheal tubes (11% vs. 5%) [13]. The novel apparatus incorporates a facial support component made of silicone material with a design tailored to facial contours, thereby increasing the support surface area for the face, consequently reducing localized pressure. After a period of prone positioning, the chest cushion, iliac crest cushion, and leg cushion of the device can be adjusted and locked, alleviating prolonged pressure on specific body support areas and thus reducing the incidence of pressure-related injuries. Bajwa et al. [14] demonstrated that approximately 59% of the patients developed facial conjunctival edema following prone ventilation. The innovative facial support structure of the new apparatus is equipped with height adjustment capabilities, which, when raised, can mitigate the occurrence of edema. During prone positioning, abdominal compression leads to increased intra-abdominal pressure, resulting in vomiting and aspiration. This apparatus suspends the patient's abdomen, reducing intra-abdominal pressure and subsequently lowering the incidence of vomiting and aspiration to 3.45%. The stable support and suspension of the head, neck, and abdomen enable the secure placement and fixation of various catheters, such as ECMO, CRRT, central venous catheters, and endotracheal tubes, preventing dislocation and obstruction. The incidence of catheter displacement or obstruction in the traditional group was 17.24%, whereas the innovative group reduced it to 3.45%.

During traditional prone ventilation, patients may exhibit intolerance or restlessness in the prone position, which can lead to various adverse events. It is critical to monitor these patients closely, especially when complex interventions such as extracorporeal life support techniques including ECMO and CRRT are being administered. However, when the novel prone ventilation apparatus is used in conjunction with extracorporeal life support techniques for critically ill patients, the incidence of catheter-related flow issues is extremely low and the longevity of CRRT filters is significantly extended. This may be attributed to the patient's suspended abdomen, reduced intra-abdominal pressure, and improved ease of maintenance and catheter patency for ECMO and CRRT lines.

Transitioning patients from the supine position to the prone position serves to re-expand dependent lung regions, thereby improving lung compliance, redistributing pulmonary edema, enhancing ventilation/perfusion ratios, and further alleviating the heterogeneity of lung pathology in ARDS patients [15]. Moreover, changes in the body position facilitate the drainage of pulmonary secretions. Prone positioning ventilation (PPV) significantly improves oxygenation in critically ill patients, but its implementation is constrained by factors such as high risk, complexity of operation, substantial manpower requirements, and the lack of specialized tools. The development of the novel prone ventilation apparatus has simplified operational procedures, reduced procedure duration, extended patient tolerance, and mitigated the risk of adverse events. To a certain extent, it has promoted the implementation and application of prone ventilation techniques. While this prone ventilation apparatus has undergone three iterations, there are still some limitations to address. Given that the novel prone positioning device has a certain height (approximately 20 cm), raising patients during the transition from the supine to the prone position still requires significant physical effort from healthcare personnel, similar to traditional prone positioning.

There are several limitations in the present study. A significant limitation of this study is the lack of registration with ClinicalTrials.gov or any other trial registry. The rapid implementation and urgent nature of the study during the height of the COVID-19 pandemic necessitated expedited research efforts to address critical clinical needs, which precluded formal registration. We acknowledge this limitation and emphasize the importance of trial registration for future research to enhance transparency and accountability. Besides, the study was conducted in a single center, which may limit the applicability of its findings to other settings with different patient demographics, clinical practices, and resources. In addition, the lack of long-term follow-up data restricts our ability to assess the enduring impacts of the novel-prone ventilation device on patient outcomes, including long-term morbidity and quality of life. Finally, the study did not account for interoperator variability in the use of the prone positioning device, which could lead to inconsistencies in patient management and outcomes. These limitations warrant further being addressed in larger, randomized, multicenter trials with standardized protocols and long-term follow-up.

The advantages of this method primarily manifest after patients have been placed in the prone position. Moving forward, improving the prone ventilation apparatus and optimizing the patient-turning process will be crucial areas of research.

In conclusion, this study presents compelling evidence for the efficacy of a novel adjustable prone position ventilation device in the management of critically ill patients requiring prone ventilation. The innovative design of the device significantly prolonged the patients' tolerance duration for prone positioning, surpassing the recommended daily minimum, and demonstrated a marked improvement in oxygen saturation levels postventilation compared to traditional methods. Furthermore, the use of the device was associated with a lower incidence of common complications such as catheter displacement, pressure injuries, and respiratory obstructions. The study also revealed the potential of the device to enhance the efficiency of extracorporeal life support technologies such as ECMO and CRRT, as indicated by the reduced frequency of suboptimal catheter blood flow and the extended lifespan of CRRT filters. These findings suggest that the novel prone position ventilation device could be a valuable tool in improving patient outcomes in critical care settings. However, the limitations inherent in the study's design, including potential measurement biases and the lack of long-term outcome data. Future studies, preferably randomized controlled trials with larger sample sizes and multicenter involvement, are essential to validate these findings and to explore the long-term impacts of this innovative approach on patient recovery and quality of life.

## Figures and Tables

**Table 1:** The basic characteristics between the two groups.

Characteristics	Traditional group (*N* = 29)	Innovative group (*N* = 29)	*t*/*χ*^2^	*P*	Difference 95% CI
Gender (*N*, male)	22 (75.86%)	21 (72.41%)	2.463	0.373	−3.45% (−16.98%, 10.08%)
Age (years)	63.5 ± 9.7	63.8 ± 9.1	3.646	0.271	0.3 (−5.4, 6.0)
BMI (kg/m^2^)	25.66 ± 5.1	26.17 ± 4.6	4.172	0.246	0.51 (−3.1, 4.1)
HR (beats/min)	90.61 ± 15.63	88.69 ± 16.76	2.673	0.527	−1.92 (−10.9, 7.1)
MAP (mmHg)	73.32 ± 11.03	73.83 ± 10.62	0.836	0.863	0.51 (−5.6, 6.6)
APACHE II score	19.21 ± 1.76	19.26 ± 2.15	0.794	0.917	0.05 (−1.4, 1.5)
SOFA score	7.52 ± 2.64	7.69 ± 2.37	0.483	0.956	0.17 (−1.4, 1.7)
Acute lung injury score	7.61 ± 3.91	8.15 ± 3.65	1.463	0.748	0.54 (−1.9, 3.0)
Lac (mmol/L)	6.31 ± 2.65	5.91 ± 3.26	4.726	0.217	−0.40 (−2.7, 1.9)
Ventilator parameters					
Tidal volume (mL)	368.49 ± 76.61	372.53 ± 79.46	3.747	0.266	4.04 (−33.4, 41.5)
Respiratory rate (times/min)	26.00 ± 6.50	26.61 ± 6.40	6.262	0.173	0.61 (−2.5, 3.7)
PEEP (cm H_2_O)	10.38 ± 4.13	11.05 ± 5.39	3.733	0.274	0.67 (−2.2, 3.5)

**Table 2:** Comparison of patient tolerance duration, increase in oxygen saturation post-PPV, and average operation duration of medical personnel between the two groups (*n* = 58).

Observation indicator	Group categories				
	Traditional group (*n* = 29)	Innovative group (*n* = 29)	*z*	*P* value	Difference 95% CI
Mean duration of prone position ventilation (h)	8.30 (5.30–9.70)	16.60 (14.10–19.60)	−1.35	<0.001	8.3 (4.4, 12.2)
Average increase in SaO_2_ (oxygen saturation) following PPV (%)	6.00 (5.00–7.00)	9.00 (8.00–11.00)	−0.33	<0.001	3.0 (1.5, 4.5)
Average duration of PPV procedure (minutes)	21.80 (20.00–25.00)	11.70 (9.00–12.00)	841.03	<0.001	−10.1 (−11.9, −8.3)

**Table 3:** Comparison of the incidence of complications during PPV between the two groups (*n* = 58).

Observation indicator	Group categories				
	Traditional group (*n* = 29)	Innovative group (*n* = 29)	*χ*2	*P* value	Difference 95% CI
Hemodynamic disturbance	2 (6.90%)	2 (6.90%)	0.01	0.982	0.00% (−10.7%, 10.7%)
Catheter displacement or occlusion	5 (17.24%)	1 (3.45%)	5.27	0.041	−13.79% (−26.8%, −0.78%)
Facial and head edema	11 (37.93%)	3 (10.34%)	6.03	0.030	−27.59% (−47.6%, −7.6%)
Pressure-related injury	12 (41.38%)	2 (6.90%)	9.42	0.005	−34.48% (−54.8%, −14.2%)
Vomiting or aspiration	5 (17.24%)	1 (3.45%)	5.27	0.041	−13.79% (−26.8%, −0.78%)

**Table 4:** Comparison of the frequency of suboptimal catheter blood flow and CRRT filter lifespan between subgroups (*n* = 20).

Observation indicator	Group categories				
	Traditional group (*n* = 10)	Innovative group (*n* = 10)	*t*/*z*	*P* value	Difference 95% CI
Catheter blood drawing (times)	69.00 ± 12.53	38.00 ± 6.37	6.97	*P* < 0.001	−31.0 (−39.6, −22.4)
CRRT filter average lifespan (h)	31.00 ± 8.48	46.00 ± 13.45	2.98	*P* < 0.001	15.0 (4.9, 25.1)

## Data Availability

The data used to support the findings of this study are available from the corresponding author upon reasonable request.

## Supplementary Materials

Supplementary Materials Appendix I: the Consolidated Standards of Reporting Trials (CONSORT) guidelines used in the present study. Appendix II: the data collection forms utilized in this study.
