# Supplementary material for: Development of Prone Position Ventilation Device and Study on the Application Effect of Combined Life Support Technology in Critically Ill Patients
Source: Can Respir J. 2024 Aug 19;2024:5812829. doi: 10.1155/2024/5812829 (PMC11347033; doi:10.1155/2024/5812829)
Supplement: Supplementary Materials — Appendix I: the Consolidated Standards of Reporting Trials (CONSORT) guidelines used in the present study. Appendix II: the data collection forms utilized in this study. [file 5812829.f1.zip › Appendix I. CONSORT_checklist_standard.docx]

# CONSORT Checklist for "Development of prone position ventilation device and study on the application effect of combined life support technology in critically ill patients"

## Title and Abstract

**1a** *Identification as a randomized trial in the title.*Title: Development of prone position ventilation device and study on the application effect of combined life support technology in critically ill patients.

**1b** *Structured summary of trial design, methods, results, and conclusions (for specific guidance see CONSORT for abstracts).*Abstract: Included in the document.

## Introduction

**2a** *Scientific background and explanation of rationale.*Background: Included in the document.

**2b** *Specific objectives or hypotheses.*Objectives: To evaluate a novel prone position ventilation device designed to enhance patient safety, improve comfort, and reduce adverse events, facilitating prolonged tolerance in critically ill patients.

## Methods

**3a** *Description of trial design (such as parallel, factorial) including allocation ratio.*Trial Design: Randomized controlled trial.

**3b** *Important changes to methods after trial commencement, with reasons.*Not applicable.

**4a** *Eligibility criteria for participants.*Participants: Critically ill patients from January 2020 to June 2023.

**4b** *Settings and locations where the data were collected.*Setting: Xuzhou Central Hospital, China.

**5** *The interventions for each group with sufficient details to allow replication, including how and when they were actually administered.*Interventions: Control group using traditional prone positioning aids vs. intervention group using a newly developed adjustable prone positioning device.

**6a** *Completely defined pre-specified primary and secondary outcome measures, including how and when they were assessed.*Outcomes: Tolerance to prone positioning, oxygen saturation increments, duration of prone positioning, CRRT filter lifespan, and incidence of adverse events.

**6b** *Any changes to trial outcomes after the trial commenced, with reasons.*Not applicable.

**7a** *How sample size was determined.*Sample Size: 60 patients (58 effective samples).

**8a** *Method used to generate the random allocation sequence.*Randomization: Computer-generated random sequence.

**8b** *Type of randomisation; details of any restriction (such as blocking and block size).*Not specified.

**9** *Mechanism used to implement the random allocation sequence (such as sequentially numbered containers), describing any steps taken to conceal the sequence until interventions were assigned.*Not specified.

**10** *Who generated the random allocation sequence, who enrolled participants, and who assigned participants to interventions.*Not specified.

**11a** *If done, who was blinded after assignment to interventions (for example, participants, care providers, those assessing outcomes) and how.*Blinding: Not specified.

**12a** *Statistical methods used to compare groups for primary and secondary outcomes.*Statistical Analysis: Data were analyzed using SPSS 26.0. Quantitative data were described with means ± standard deviation or median (interquartile range) where appropriate. Categorical data were expressed as frequency (%). The t-test or Mann-Whitney U test was used for mean comparisons and the chi-square or Fisher's exact test for categorical data. Significance was set at P < 0.05 and high significance at P < 0.01.

## Results

**13a** *For each group, the numbers of participants who were randomly assigned, received intended treatment, and were analyzed for the primary outcome.*Participant Flow: Diagram not provided in the document, but patient distribution and outcomes are described.

**13b** *For each group, losses and exclusions after randomisation, together with reasons.*Exclusions: One patient self-discharged during treatment, and another was terminated due to decreased oxygenation.

**14a** *Dates defining the periods of recruitment and follow-up.*Recruitment: Conducted from January 2020 to June 2023.

**15** *A table showing baseline demographic and clinical characteristics for each group.*Baseline Data: Provided in Table 1.

**16** *For each group, number of participants (denominator) included in each analysis and whether the analysis was by original assigned groups.*Numbers Analyzed: 58 patients (29 in each group).

**17a** *For each primary and secondary outcome, results for each group, and the estimated effect size and its precision (such as 95% confidence interval).*Outcomes and Estimation: Detailed results provided, including statistical significance and confidence intervals.

**18** *Results of any other analyses performed, including subgroup analyses and adjusted analyses, distinguishing pre-specified from exploratory.*Not specified.

**19** *All important harms or unintended effects in each group (for specific guidance see CONSORT for harms).*Harms: Detailed in the results section.

## Discussion

**20** *Trial limitations, addressing sources of potential bias, imprecision, and, if relevant, multiplicity of analyses.*Limitations: Single-center study, lack of long-term follow-up, no registration with ClinicalTrials.gov.

**21** *Generalisability (external validity, applicability) of the trial findings.*Generalisability: Limited by single-center study and lack of long-term follow-up.

**22** *Interpretation consistent with results, balancing benefits and harms, and considering other relevant evidence.*Interpretation: The new device improves tolerance to prone positioning, enhances oxygenation, and minimizes adverse events.

## Other Information

**23** *Registration number and name of trial registry.*Registration: Not registered with ClinicalTrials.gov.

**24** *Where the full trial protocol can be accessed, if available.*Protocol: Ethical approval obtained, protocol described.

**25** *Sources of funding and other support (such as supply of drugs), role of funders.*Funding: Supported by the Xuzhou City Science and Technology Plan Project (KC21202).
