# Supplementary material for: Development of Prone Position Ventilation Device and Study on the Application Effect of Combined Life Support Technology in Critically Ill Patients
Source: Can Respir J. 2024 Aug 19;2024:5812829. doi: 10.1155/2024/5812829 (PMC11347033; doi:10.1155/2024/5812829)
Supplement: Supplementary Materials — Appendix I: the Consolidated Standards of Reporting Trials (CONSORT) guidelines used in the present study. Appendix II: the data collection forms utilized in this study. [file 5812829.f1.zip › Appendix II. Data Collection Form for Prone Ventilation Study.docx]

**Appendix II. Data Collection Form for Prone Ventilation Study**

**Section 1: Patient Demographics**

Patient ID: [________]

Date of Admission: [ ]

Age: [________]

Gender: [Male/Female/Other]

Weight (kg): [________]

Height (cm): [________]

**Section 2: Clinical Background**

Diagnosis:

- [ ] ARDS

- [ ] COVID-19-induced ARDS

- [ ] Other: [Specify________]

Comorbidities (Check all that apply):

- [ ] Hypertension

- [ ] Diabetes

- [ ] Cardiovascular Disease

- [ ] None

- [ ] Other: [Specify________]

**Section 3: Ventilation Details**

Ventilation Start Date: [ ]

Type of Ventilation:

- [ ] Conventional Mechanical Ventilation

- [ ] Prone Position Ventilation

Ventilator Settings:

PEEP (cm H2O): [________]

FiO2 (%): [________]

Tidal Volume (mL): [________]

Respiratory Rate (per min): [________]

**Section 4: Extracorporeal Support (If Applicable)**

Use of ECMO:

- [ ] Yes

- [ ] No

Use of CRRT:

- [ ] Yes

- [ ] No

**Section 5: Monitoring and Outcomes**

Daily Monitoring:

Oxygen Saturation (SaO2) (%): [________]

Blood Pressure (mmHg): [Systolic________/Diastolic________]

Heart Rate (beats per min): [________]

Temperature (°C): [________]

Complications (Check all that apply and specify date of occurrence):

- [ ] Pressure Injuries (Date: [ ])

- Location and Severity: [Specify________]

- [ ] Catheter Migration (Date: [ ])

- [ ] Vomiting/Aspiration (Date: [ ])

- [ ] Hemodynamic Disturbance (Date: [ ])

- [ ] None

- [ ] Other: [Specify________]

**Section 6: Outcome**

Date of Ventilation Cessation: [ ]

Overall Outcome:

- [ ] Recovered

- [ ] Transferred to another facility

- [ ] Deceased (Date of Death: [ ])

Notes/Observations: [ ]

**Section 7: Data Collector Information**

Collected By: [ ]

Date of Collection: [ ]

Signature: [________]
